# Supplementary material for: Healing of Experimental Periodontal Defects Following Treatment with Fibroblast Growth Factor-2 and Deproteinized Bovine Bone Mineral
Source: Biomolecules. 2021 May 29;11(6):805. doi: 10.3390/biom11060805 (PMC8226676; doi:10.3390/biom11060805)
Supplement: Supplementary file 1 [file biomolecules-11-00805-s001.zip › biomolecules-1230513-supplementary.pdf]

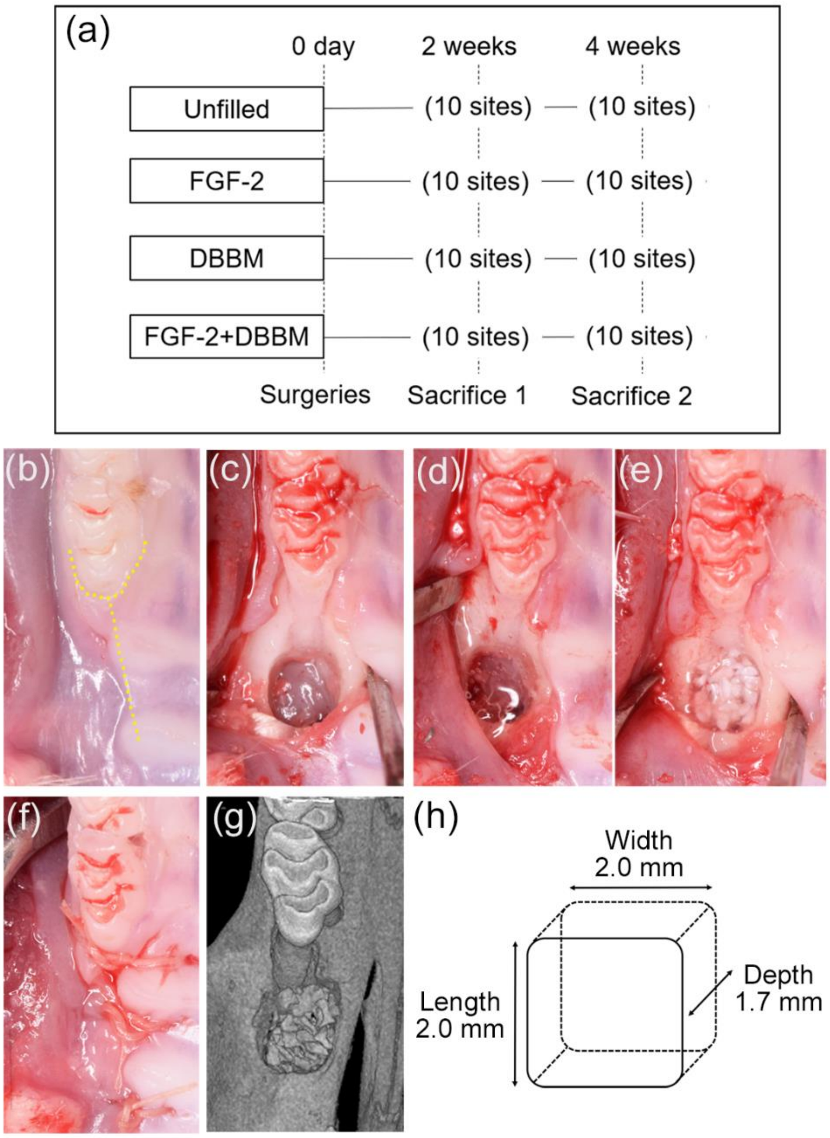


**Figure S1**. *In vivo* experimental protocol and representative images of surgical creation of periodontal defects (FGF-2+DBBM group).

(a) Experimental protocol and groups (b) Incision design (c) After raising full-thickness flaps, bilateral standardized periodontal defects (2.0 × 2.0 × 1.7 mm) were created mesially of the maxillary first molars (M1). (d) Application of FGF-2 mixed with HPC. (e) Application of DBBM (pre-mixed with FGF-2). (f) Flaps were closed using resorbable sutures. (g) Micro-CT image of the periodontal defect immediately after surgery. (h) Standardized defect size.

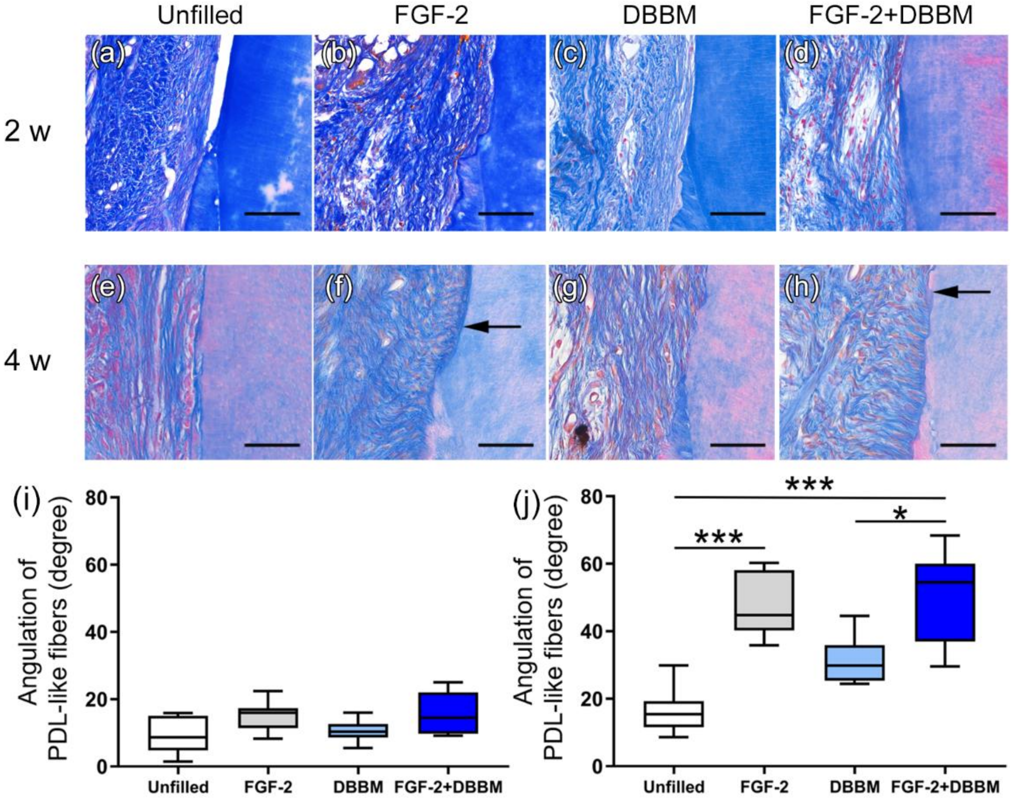


**Figure S2**: Healing of periodontal ligament (PDL)

(a-h) Representative photomicrographs of the root area near the bottom of the defect at 2 and 4 weeks postoperatively. (a-d) At 2 weeks postoperatively (upper panel), collagen bundles run near parallel to the surfaces in all groups. (f, h) At 4 weeks postoperatively, PDL-like collagen bundles are well-aligned and obliquely inserted onto the root surface similar to native PDL with signs of thin-layer of cementogenesis (indicated by an arrow) in the FGF-2 and FGF-2+DBBM groups. (Azan-Mallory's stain, original magnification ×200; bar = 50 μm). (i, j) Angulation of the PDL-like fibers. The angulation of fiber bundles at the bottom area of instrumentation on M1 root was observed under ×200 and analyzed by Image J software at 2 weeks (i) and 4 weeks (j) postoperatively. Box in white, Unfilled group; box in gray, FGF-2 group; box in light blue, DBBM group; box in blue, FGF-2+DBBM group. Data shown as box-and-whiskers plot with minimum, maximum, median, and 25th and 75th percentiles (n = 6) in degree. $*p < 0.05$, $***p < 0.001$ by ANOVA with Tukey post-test.

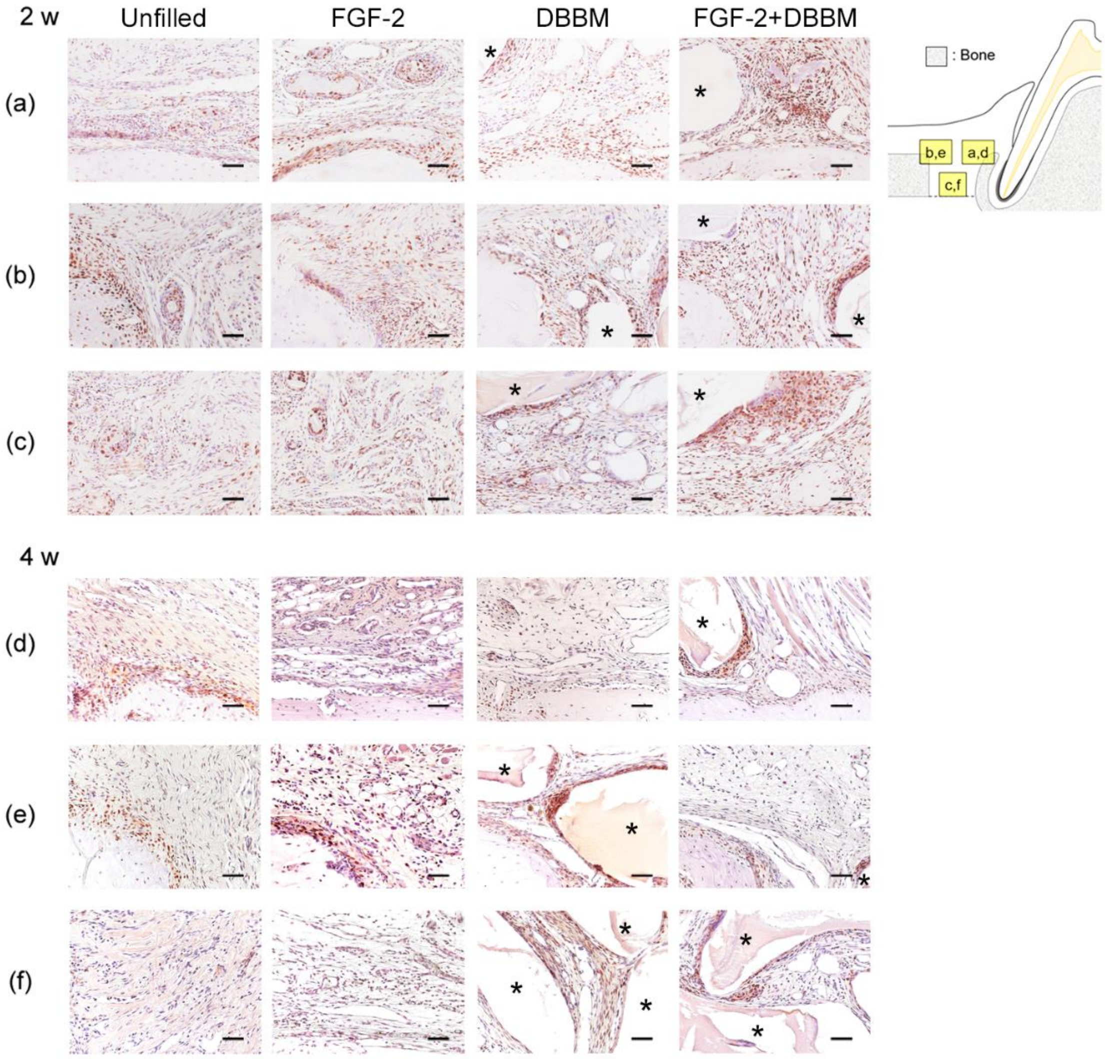


**Figure S3**: Representative photomicrographs of immunohistochemical staining for PCNA

Prevalence of PCNA-positive cells is assessed in Root side (a,d), Bone side (b,e), and middle area (c,f). A brown coloration indicates a PCNA-positive reaction. At 2 weeks (a-c), the number of PCNA-positive cells in all areas appears to be greater in the FGF-2 and FGF-2+DBBM groups compared with Unfilled groups. At 4 weeks (d-f), no obvious differences are observed among groups. (PCNA and counterstaining with Mayer's hematoxylin stain, original magnification ×200; bar = 50 µm, Asterisk indicates DBBM particles)

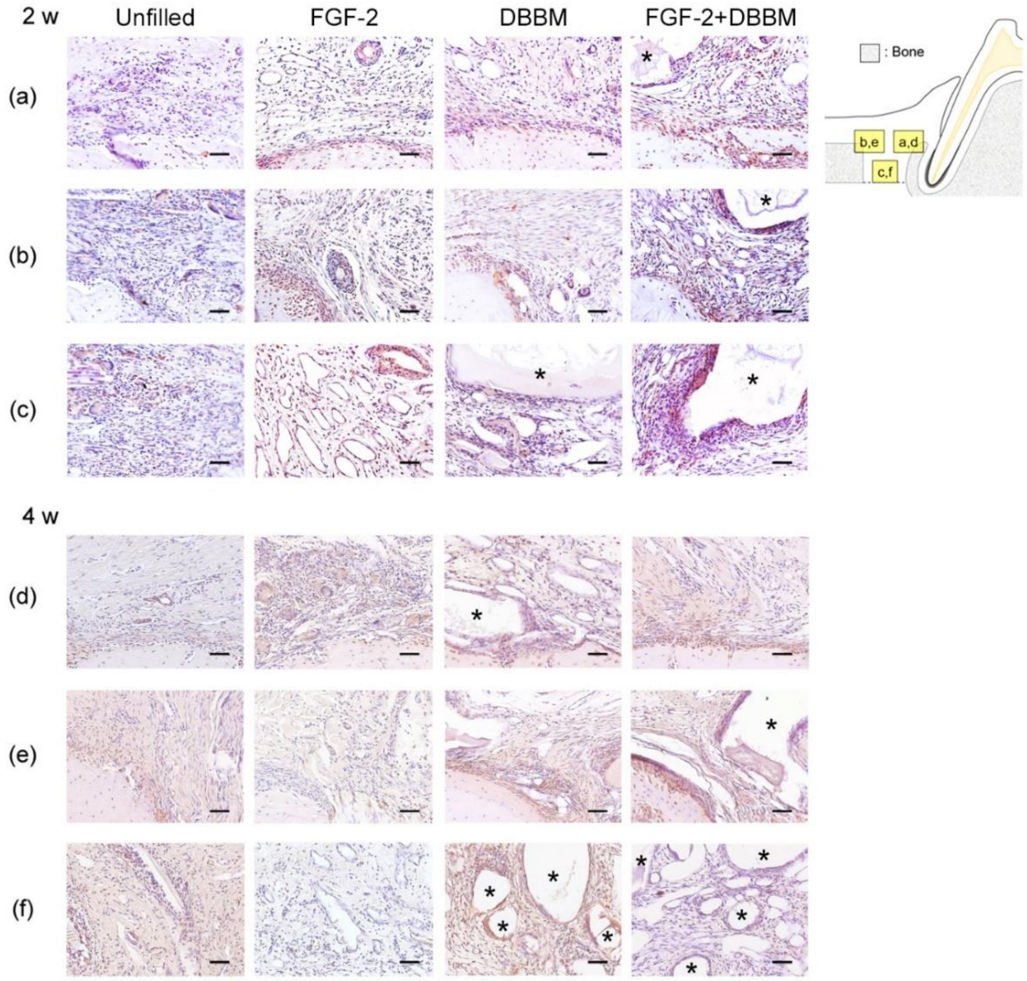


**Figure S4**: Representative photomicrographs of immunohistochemical staining for VEGF.

Prevalence of VEGF-positive cells is assessed in Root side (a,d), Bone side (b,e), and Middle area (c,f). A brown coloration indicates a VEGF-positive reaction. At 2 weeks (a-c), the prevalence of VEGF-positive cells in all areas in the FGF-2 and FGF-2+DBBM groups appears to be greater than in the Unfilled group. At 4 weeks (d-f), no obvious differences are observed among groups. (VEGF and counterstaining with Mayer's hematoxylin stain, original magnification ×200; bar = 50 µm, Asterisk indicates DBBM particles)

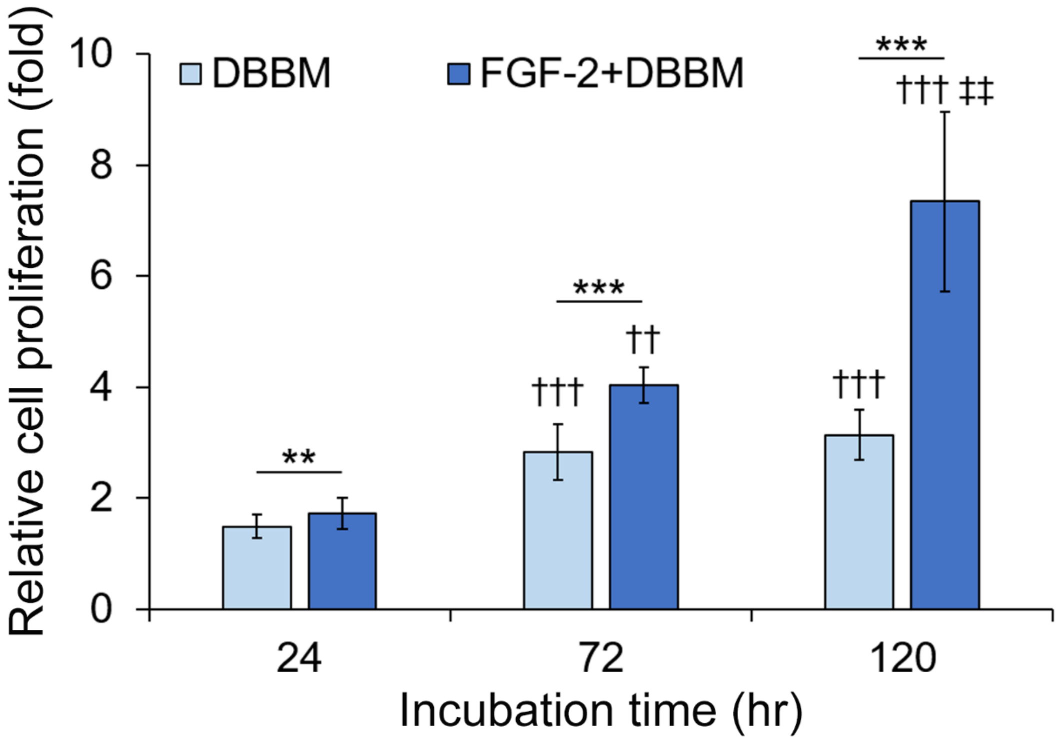


**Figure S5**: *In vitro* viability/proliferation of PDLCs.

PDLCs were seeded onto the DBBM with/without FGF-2 and allow to grow for up to 120 hr. WST-1 assay was used to determine cell viability/proliferation at indicated time points. The reference absorbance at 450 nm was subtracted from the absorbance for each sample, and the values relative to those at 0 hr were shown. PDLCs on the FGF-2 treated DBBM showed significantly higher viability/proliferation than the DBBM at 24 hr, 72 hr, and 120 hr. Data shown as mean ± SD (n = 8). ** $p < 0.01$, *** $p < 0.001$ by Mann-Whitney U test. †† $p < 0.01$, ††† $p < 0.001$ significant difference from 24 hr values and ‡‡ $p < 0.01$ significant difference from 72 hr values by Friedman test with Dunn's post-test.
